# Lysine demethylase 2A promotes stemness and angiogenesis of breast cancer by upregulating Jagged1

**DOI:** 10.18632/oncotarget.8381

**Published:** 2016-03-25

**Authors:** Jing-Yi Chen, Chien-Feng Li, Pei-Yi Chu, You-Syuan Lai, Chung-Hsing Chen, Shih Sheng Jiang, Ming-Feng Hou, Wen-Chun Hung

**Affiliations:** ^1^ National Institute of Cancer Research, National Health Research Institutes, Tainan 704, Taiwan; ^2^ Department of Pathology, Chi-Mei Foundation Medical Center, Tainan 710, Taiwan; ^3^ School of Medicine, College of Medicine, Fu-Jen Catholic University, New Taipei City 242, Taiwan; ^4^ Department of Pathology, Show Chwan Memorial Hospital, Changhua City 500, Taiwan; ^5^ Department of Surgery, College of Medicine, Kaohsiung Medical University, Kaohsiung 807, Taiwan; ^6^ Cancer Center, Kaohsiung Medical University Hospital, Kaohsiung 807, Taiwan; ^7^ Graduate Institute of Medicine, College of Medicine, Kaohsiung Medical University, Kaohsiung 807, Taiwan

**Keywords:** lysine demethylase 2A, angiogenesis, stemness, Jagged1, SOX2

## Abstract

Alterations of histone methylation dynamically regulated by methyltransferases and demethylases are frequently found in human cancers. Here, we showed that expression of lysine demethylase 2A (KDM2A) is markedly increased in human breast cancer and its overexpression is associated with tumor progression and poor prognosis. Knockdown of KDM2A in breast cancer cells reduced proliferation but not viability. Gene set enrichment analysis revealed that inhibition of KDM2A down-regulates angiogenic genes with concurrent reduction of Jagged1 (JAG1), NOTCH1 and HEY1 in the NOTCH signaling. Chromatin immunoprecipitation- quantitative polymerase chain reaction (ChIP-qPCR) demonstrated the binding of KDM2A to the JAG1 promoter and the increase of methylation of Lys-36 of histone H3 (H3K36) in KDM2A-depleted MDA-MB-231 cells. Tumorsphere formation was significantly reduced in KDM2A-depleted cells which could be reversed by ectopic expression of JAG1. A selective KDM2A inhibitor daminozide also decreased the number of tumorsphere and the number of CD24^−^/CD44^hi^ cells. In addition, daminozide acted synergistically with cisplatin in cell killing. We identified SOX2 as a direct transcriptional target of KDM2A to promote cancer stemness. Depletion of KDM2A in MDA-MB-231 cells attenuated NOTCH activation and tube formation in co-cultured endothelial cells. Two pro-angiogenic factors JAG1 and PDGFA are key mediators for KDM2A to enhance angiogenesis. Finally, inhibition of KDM2A significantly decreased tumor growth and angiogenesis in orthotopic animal experiments. Collectively, we conclude that KDM2A functions as an oncogene in breast cancer by upregulating JAG1 to promote stemness, chemoresistance and angiogenesis.

## INTRODUCTION

Histone proteins are the building components of the nucleosome, the basic unit of DNA packaging in eukaryotic cells. Histone proteins undergo different post-translational modifications (PTMs) including phosphorylation, acetylation, methylation, sumoylation and ubiquitination. These modifications affect the compact of chromatin structure and the interactions between non-histone proteins and chromatin to modulate gene expression. The ultimate effects of histone modification on gene transcription dependent on the type of modification, the modified residues, and the degree of modification [1–3]. Tri-methylation of lysine 4 of histone H3 (H3K4) is strongly associated with transcriptional activation and is frequently found around the transacription start sites of highly expressed genes, whereas H3K27 tri-methylation is usually linked with transcriptional inhibition and is detected in the promoters of silenced genes [4–6]. In contrast to the repressive role of H3K27 tri-methylation, recent study suggested that mono-methylated H3K27 accumulates within active genes and promotes transcriptional activation [7].

Methylation of histone proteins is tightly regulated by histone methyltransferases and demethylases [8]. Lysine demethylase 2A (KDM2A) was originally cloned as a member of mammalian F-box protein families that are critical components of the SCF ubiquitin-protein ligase complexes [9]. Subsequently, another group cloned a novel gene CXXC8 (also known as FBXL11) as a new member of CXXC family genes [10]. Tsukada et al purified a JmjC domain-containing protein JHDM1 which acts as a H3K36 demethylase by using Fe2+ and α-ketoglutarate as cofactors [11]. It turns out that these three molecules are encoded by the same gene, and is now officially named as KDM2A. Among the methylation sites of histone H3 studied, the biological significance of H3K36 is relatively unclear. H3K36 methylation has been associated with transcription activation. However, the genetic location where the methylated H3K36 is added, the timing when H3K36 is methylated, and the degree of methylation all determine the final biological outcomes [12].

The potential role of KDM2A in carcinogenesis has been demonstrated recently in lung cancer [13]. KDM2A was identified as one of the most upregulated histone demethylases in lung cancer. This demethylase induced H3K36 demethylation of the promoter region of the dual-specificity phosphatase 3 (DUSP3) gene that led to down-regulation of DUSP3. Because DUSP3 is an important dephsophorylating enzyme of extracellular signal-regulated kinases (ERKs), reduced expression of DUSP3 increased ERK activation and promoted cell proliferation and invasiveness. Results of the study suggested KDM2A is an oncogene in lung cancer. However, the contribution of KDM2A to other cancers is largely unknown. In addition, the downstream mediators by which KDM2A promotes carcinogenesis is also unclear. In the present study, we investigated the expression of KDM2A in breast cancer tissues and examined its association with clinicopathological features. In addition, we tried to elucidate how KDM2A regulates the behaviors of breast cancer cells and identified JAG1 as a key downstream effector for KDM2A to promote stemness and angiogenesis in breast cancer.

## RESULTS

### Upregulation of KDM2A in breast cancer is associated with short survival

**Figure 1:**
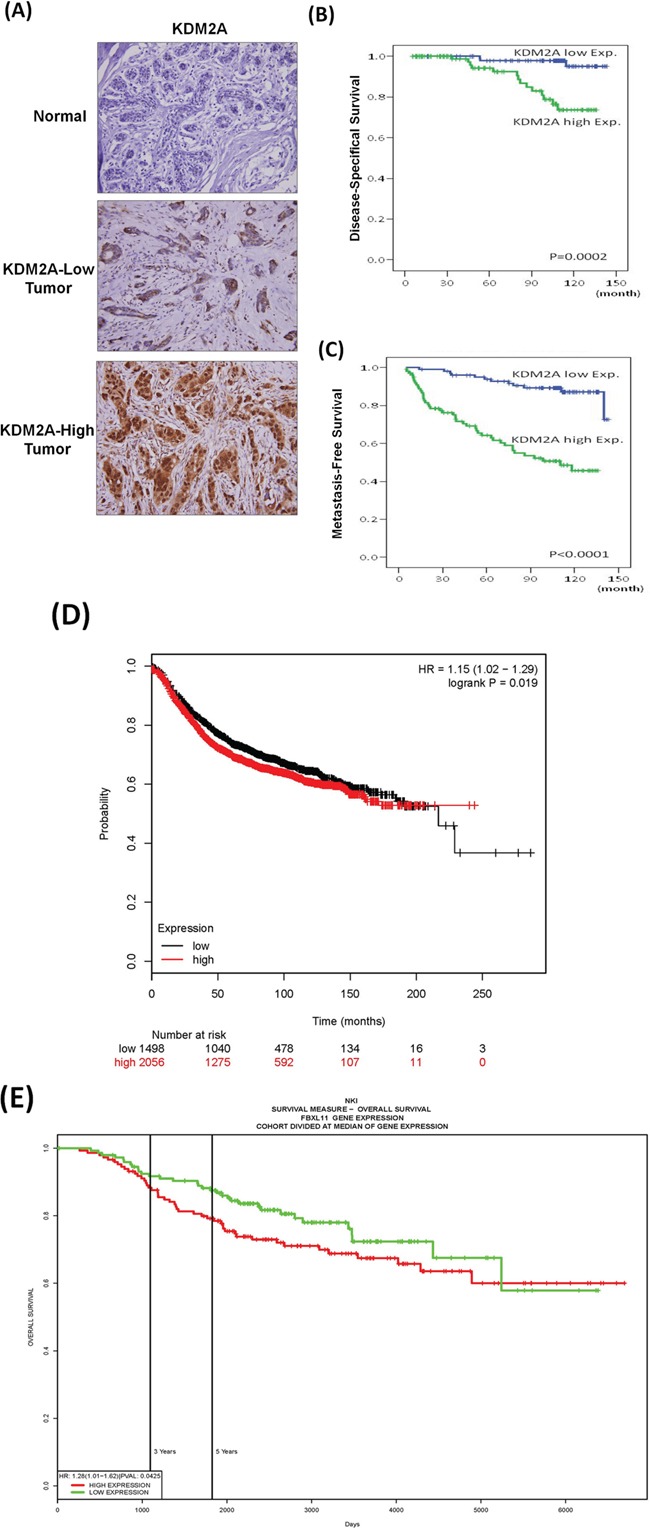
High expression of KDM2A is associated short survival of breast cancer patients **A.** Immunohistochemical staining of KDM2A in normal and tumor tissues of breast cancer. **B.** Disease-free survival of 202 breast cancer patients with high or low KDM2A expression. **C.** Metastasis-free survival of 202 breast cancer patients with high or low KDM2A expression. **D.** KDM2A expression is associated with poor survival (hazard ratio=1.15, *p*=0.019) in breast cancer patients analyzed by the KM Plotter database. **E.** KDM-high breast cancer patients have a lower overall survival analyzed by the PROGgeneV2 database (*p*=0.0425).

We performed immunohistochemical staining to investigate the expression of KDM2A in 202 breast tumor tissues. As shown in Figure 1A, fifty percent (101/202) of tumor tissues exhibited high expression of KDM2A with strong nuclear staining and light cytoplasmic staining. Clinicopathological association study demonstrated that high KDM2A is significantly correlated with large primary tumor, increased lymph node metastasis, advanced stage and high histological grade (Table 1). Kaplan-Meier survival analysis demonstrated that patients with high KDM2A expression have a short disease-specific survival (Figure 1B) and metastasis-free survival (Figure 1C). Univariate log-rank analysis showed tumor size, nodal status, stage and KDM2A expression are associated with the survival of breast cancer patients (Table 2). In addition, high KDM2A expression appeared as an independent prognostic factor for disease-specific survival (hazard ratio=5.406, 95% CI=1.447 to 20.201, P=0.012) and metastasis-free survival (hazard ratio=3.476, 95% CI=1.727 to 6.998, P<0.001) in multivariate analysis (Table 3). These data suggested that breast cancer patients with KDM2A up-regulation have poor prognosis. To validate our conclusion, we did bioinformatics analysis by using the public databases of PM Plotter [14] and PROGgeneV2 [15]. By using the Jetset best probe #208988 and auto select cutoff value for analysis, high KDM2A expression is associated poor survival (hazard ratio=1.15 (1.02-1.29) and *p*=0.019) (Figure 1D). Similarly, the PROGgeneV2 database indicated a poor overall survival of breast cancer patients with high KDM2A expression (*p*=0.0425) (Figure 1E).

**Table 1:** Correlation between KDM2A expression and various clinicopathological factors

Parameters	Category	No. of case	KDM2A Expression		*P*-valuve
			Low	High	
**Age (years)**	<60 years	141	67	74	0.283
	≧60 years	61	34	27	
**Primary tumor (T)**	T1	82	52	30	<0.001^*^
	T2	96	47	49	
	T3-4	24	2	22	
**Nodal status (N)**	N0	117	81	36	<0.001^*^
	N1-N2	85	20	65	
**Stage**	I	63	45	18	<0.001^*^
	II	115	55	60	
	III	24	1	23	
**Histological grade**	Grade I	18	14	4	0.034^*^
	Grade II	141	69	72	
	Grade III	43	18	25	

* **Statistically significant**

**Table 2:** Univariate log-rank analysis for disease-specific survival and metastasis-free survival

Parameters	Category	No. of case	DSS		MeFS	
			No. of event	*P*-value	No. of event	*P*-value
**Age (years)**	<60 years	141	13	0.9804	41	0.7650
	≧60 years	61	4		16	
**Primary tumor (T)**	T1	82	5	0.0283^*^	9	<0.0001^*^
	T2	96	10		34	
	T3-4	24	2		14	
**Nodal status (N)**	N0	117	7	0.0079^*^	19	<0.0001^*^
	N1-2	85	10		38	
**Stage**	I	63	3	0.0001^*^	5	<0.0001^*^
	II	115	10		38	
	III	24	4		14	
**Histological grade**	Grade I	18	0	0.2066	1	0.0269^*^
	Grade II	141	15		41	
	Grade III	43	2		15	
**KDM2A expression**	Low Exp.(<medium)	101	3	0.0002^*^	12	<0.0001^*^
	High Exp.(≧medium)	101	14		45	

* **Statistically significant**

**Table 3:** Multivariate survival analysis

Parameter	Category	DSS			MeFS		
		H.R	95% CI	*P*-value	H.R	95% CI	*P*-value
**Stage**	I	1	−	0.050^*^	1	-	**<0.001**
	II-III	1.793	0.480-6.703		4.572	1.596-13.097	
	III	5.143	1.052-25.129		11.234	3.496-36.098	
**KDM2A expression**	Low Exp.(<medium)	1	-	0.012^*^	1	-	**<0.001**
	High Exp.(≧medium)	5.406	1.447-20.201		3.476	1.727-6.9976	
**Histological grade**	Grade I	-	-	-	1	-	0.274
	Grade II	-	-	-	3.508	0.470-26.175	
	Grade III	-	-	-	4.693	0.603-36.552	

* **Statistically significant**

### Inhibition of KDM2A induces G1 progression delay and reduces proliferation in breast cancer cells

**Figure 2:**
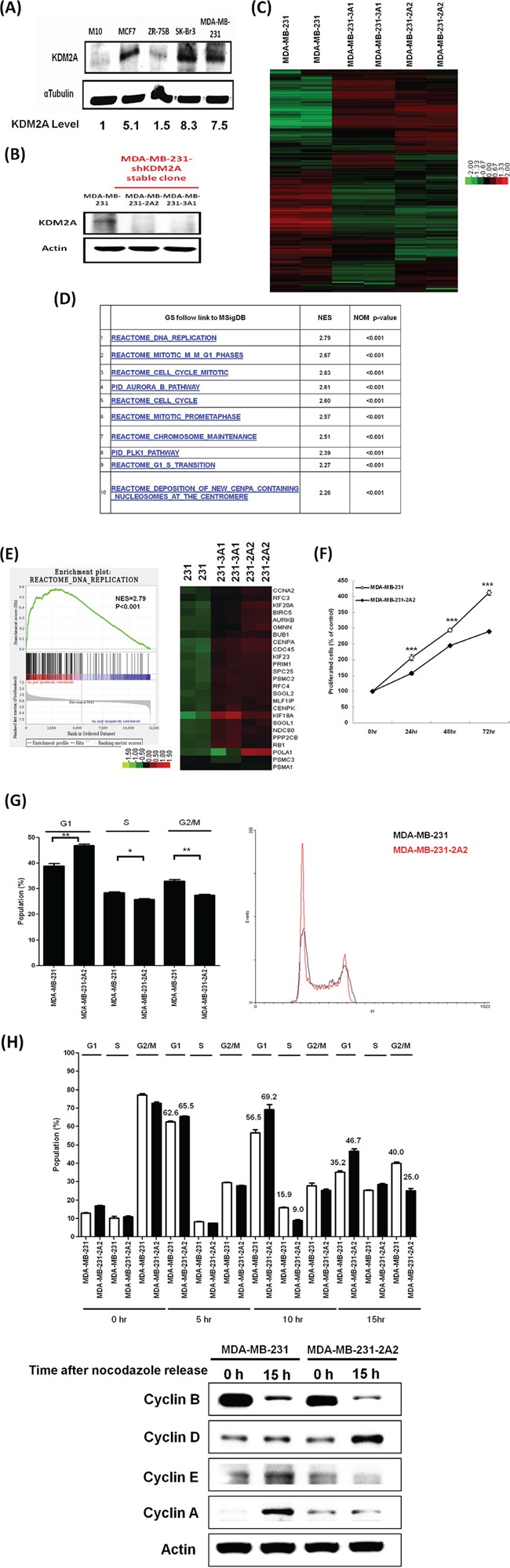
Knockdown of KDM2A delayed cell cycle progression and decreased proliferation of breast cancer cells **A.** The protein level of KDM2A in various breast cancer cell lines was determined by Western blot analysis. **B.** MDA-MB-231 cells were transfected with different shRNA and two stable clones were established by antibiotic selection. The KDM2A protein level was determined by Western blot analysis. **C.** Gene expression profiles of MDA-MB-231 and KDM2A-depleted stable cell lines. **D.** Top 10 differentially expressed gene pathways in KDM2A-depleted stable cell lines. **E.** GSEA analysis showed the core DNA replication genes changed in two KDM2A-depleted clones (left panel) and the core genes in the pathway were shown (right panel). **F.** Theproliferation of KDM2A-dpleted MDA-MB-231-2A2 cells and parental MDA-MB-231 cells was studied by MTT assaya. **G.** A typical flow cytometry analysis demonstrated the increase of cells at the G0/G1 phase and the decrease of cells at the S and G2/M phases in KDM2A-depleted MDA-MB-231-2A2 cells (right panel). Results from three independent assays were shown in the left panel. **H.** Cells were synchronized at the G2/M phase by nocodazole. Drug was washed out after 24 h and cells were harvested at 5, 10 and 15 h after release for cell cycle analysis. *p<0.05, **p<0.01 and ***p<0.001. The cells at 0 and 15 h after nocodazole release were also harvested for Western blot analysis and the expression of cyclins was studied by Western blot analysis.

We next investigated the expression of KDM2A in breast cancer cell lines. As shown in Figure 2A, KDM2A protein level is very low in normal human mammary epithelial M10 cells and a 5.1-, 8.3-, and 7.5-fold increase was detected in MCF-7, SkBr3 and MDA-MB-231 cells respectively. To investigate the effect of KDM2A on cancer cell behaviors, we generated two independent KDM2A-depleted stable clones (231-3A1 and 231-2A2) from MDA-MB-231 cells by shRNA knockdown (Figure 2B). Gene expression profiles of two KDM2A-depleted clones are distinct from that of parental cells (Figure 2C). Gene set enrichment assay (GSEA) analysis indicated that the most significantly changed pathways are related to DNA replication and cell cycle regulation (Figure 2D). Interestingly, in the top 25 core genes in the DNA replication geneset obtained by GSEA, both cell cycle promoters (like *CCNA2*, *CDC45* and *AURKB*) and inhibitors (like *RB1*, *GMNN* and *PPP2CB*) are upregulated in KDM2A-depleted cells (Figure 2E). Therefore, the effect of KDM2A depletion in breast cancer cells was further investigated. We found that the proliferation of MDA-MB-231-2A2 cells was reduced (Figure 2F). The doubling time of MDA-MB-231 cells is 23.1 h while it increased to 36.8 h in MDA-MB-231-2A2 cells. Flow cytometry analysis revealed the increase of cells at the G0/G1 phase and a reduction of cells at the S and G2/M phases (Figure 2G). Because the decrease of S-phase cells is minor, we thought KDM2A depletion delays the G1 progression but not completely blocks cell cycle transition. To test our hypothesis, we synchronized cells at the G2/M phase by nocodazole and then released the cells for cell cycle analysis at different times. At 5 h after nacodazole release, the percentage of cells at the G1 phase is similar in MDA-MB-231 (62.6%) and MDA-MB-231-2A2 (65.5%) cells (Figure 2H). At 10 h, the percentage of the G1 phase of MDA-MB-231 cells was 56.5% while it increased to 69.2% in MDA-MB-231-2A2 cells. At 15 h, 35.2% of MDA-MB-231 cells and 46.7% of MDA-MB-231-2A2 cells were at the G1 phase (Figure 2H). I harvested the cells and detected the level of G1 and S phase cyclins. As shown in the low panel of Figure 2H, treatment of nocodazole inhibited cell cycle at the G2/M phase with a dramatic increase of the mitotic cyclin B. After releasing nocodazole inhibition for 15 h, cyclin B was reduced in MDA-MB-231 cells and two S phase cyclins (cyclin E and A) were significantly increased indicating these cells entered the S phase. Conversely, MDA-MB-231-2A2 cells expressed high level of cyclin D and very low level of cyclin E and A suggesting these cells were still at the G1 phase. These results supported our notion that simultaneous upregulation of cell cycle promoters and inhibitors in KDM2A-depleted cells delays the G1 progression and decreases cell proliferation.

### KDM2A knockdown attenuates JAG1 expression and NOTCH1 activation

**Figure 3:**
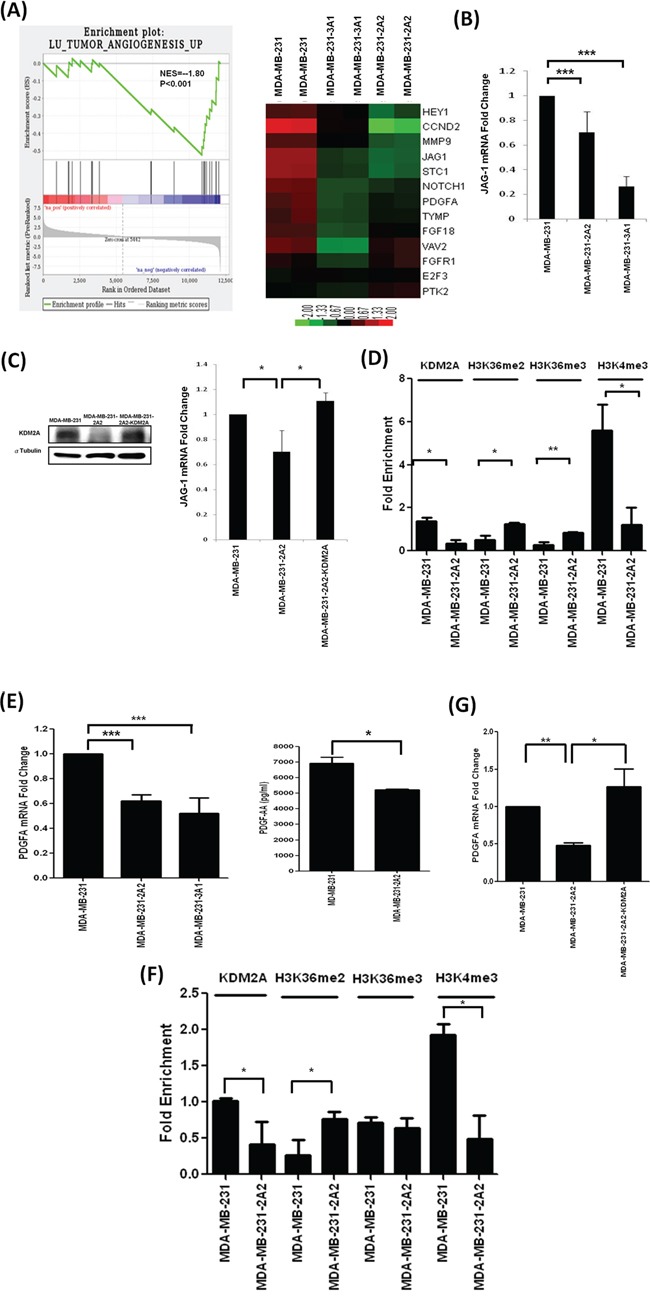
Angiogenesis gene pathway and JAG1 were down-regulated in KDM2A-depleted cells **A.** GSEA analysis demonstrated the downregulation of angiogenesis gene pathway and the concurrent decrease of *JAG1*, *NOTCH1* and *HEY1* in KDM2A-depleted cells. **B.** Total RNA was harvested from MDA-MB-231 cells and two KDM2A-depleted clones. The expression of *JAG1* mRNA was quantified by qRT-PCR. **C.** The mRNA and protein levels of KDM2A in breast cancer cell lines with KDM2A knockdown or overexpression were studied by Western blot analysis and qRT-PCR. **D.** Quantitative ChIP-PCR showed the decrease of KDM2A binding to the *JAG1* promoter and the alteration of histone methylation status in proximal promoter region in KDM2A-depleted cells. **E.** The expression of *PDGFA* mRNA in MDA-MB-231 and two KDM2A-depleted stable clones was investigated by qRT-PCR. The amount of PDGF-AA released into the conditioned medium was determined by ELISA assay. **F.** The binding of KDM2A to *PDGFA* promoter and the methylation status of *PDGFA* promoter were studied by ChIP assay combined with q-PCR determination. **G.** Ectopic expression of KDM2A in the KDM2A-depleted MDA-MB-231-2A2 cells reversed the reduction of *PDGFA* mRNA. *p<0.05, **p<0.01 and ***p<0.001.

In addition to alteration in cell proliferation, our GSEA analysis demonstrated that inhibition of KDM2A attenuates tumor angiogenesis and mRNA expression of several genes in the NOTCH signaling pathway including *JAG1*, *NOTCH1*, *HEY1* (Figure 3A). Because JAG1 is the ligand for NOTCH1, we investigated whether KDM2A depletion reduces *JAG1* expression and found that it is indeed the case (Figure 3B). Ectopic expression of KDM2A in MDA-MB-231-2A2 cells fully rescued the downregulation of JAG1 indicating KDM2A is an upstream regulator of JAG1 (Figure 3C). In addition, ChIP-qPCR assay demonstrated that KDM2A directly bound to the *JAG1* promoter and the binding was significantly reduced in MDA-MB-231-2A2 cells (Figure 3D). Consequently, di-methylation and tri-methylation of hisone H3 lysine-36 (H3K36me2 and H3K36me3) in the *JAG1* promoter is increased. In consistent with the reduction of JAG1 expression, the gene activation marker H3K4 was significantly decreased (Figure 3D). We found that PDGFA is also a direct transcriptional target of KDM2A. The mRNA level of PDGFA and the secreted PDGFA protein were reduced in KDM2A-depleted cells (Figure 3E). ChIP-qPCR assay demonstrated the direct binding of KDM2A to the *PDGFA* promoter (Figure 3F). In KDM2A-depelted cells, di-methylation of H3K36 of the *PDGFA* promoter was increased and the gene activation marker H3K4 was decreased (Figure 3F). Additionally, ectopic expression of KDM2A reversed *PDGFA* expression in KDM2A-depleted cells (Figure 3G).

**Figure 4:**
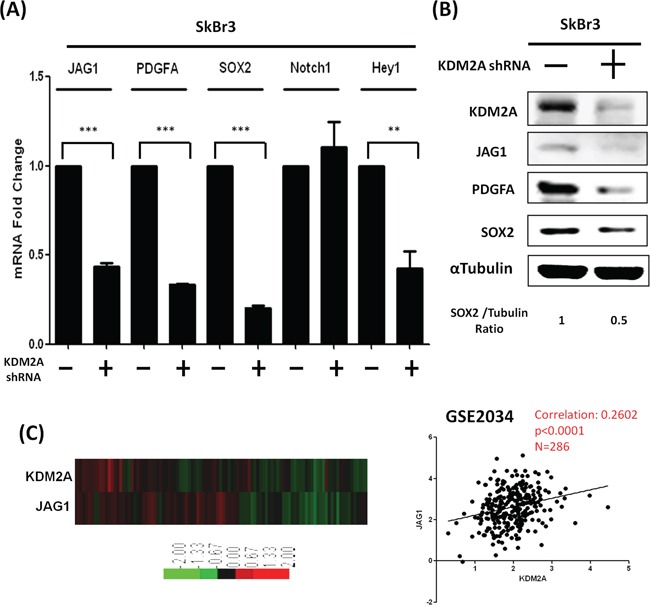
Knockdown of KDM2A also reduced JAG1 and PDGFA in SkBr3 breast cancer cells **A.** Expressions of different target genes in SkBr3 cells transfected with control or KDM2A shRNA were studied by qRT-PCR. **B.** Western blot analysis was performed to demonstrate the protein level of various target genes in control and KDM2A-depleted SkBr3 cells. **C.** KDM2A expression is positively associated with JAG1 in a dataset containing the results of 286 breast cancer patients. *p<0.05, **p<0.01 and ***p<0.001.

To rule out the cell line-specific effect, we inhibited KDM2A in SkBr3 breast cancer cells and found the expression of *JAG1* and *PDGFA* was also reduced (Figure 4A and 4B). To confirm the clinical relevance, we performed bioinformatics analysis of a public database (GSE2034) with the gene expression profiles of 286 breast cancer patients. We found a strong positive correction (*P*<0.0001) between *KDM2A* and *JAG1* in these cancer patients (Figure 4C). These data suggested that *JAG1* is a direct target of KDM2A to promote the activation of NOTCH1.

### KDM2A increases stemness and chemoresistance of breast cancer cells

**Figure 5:**
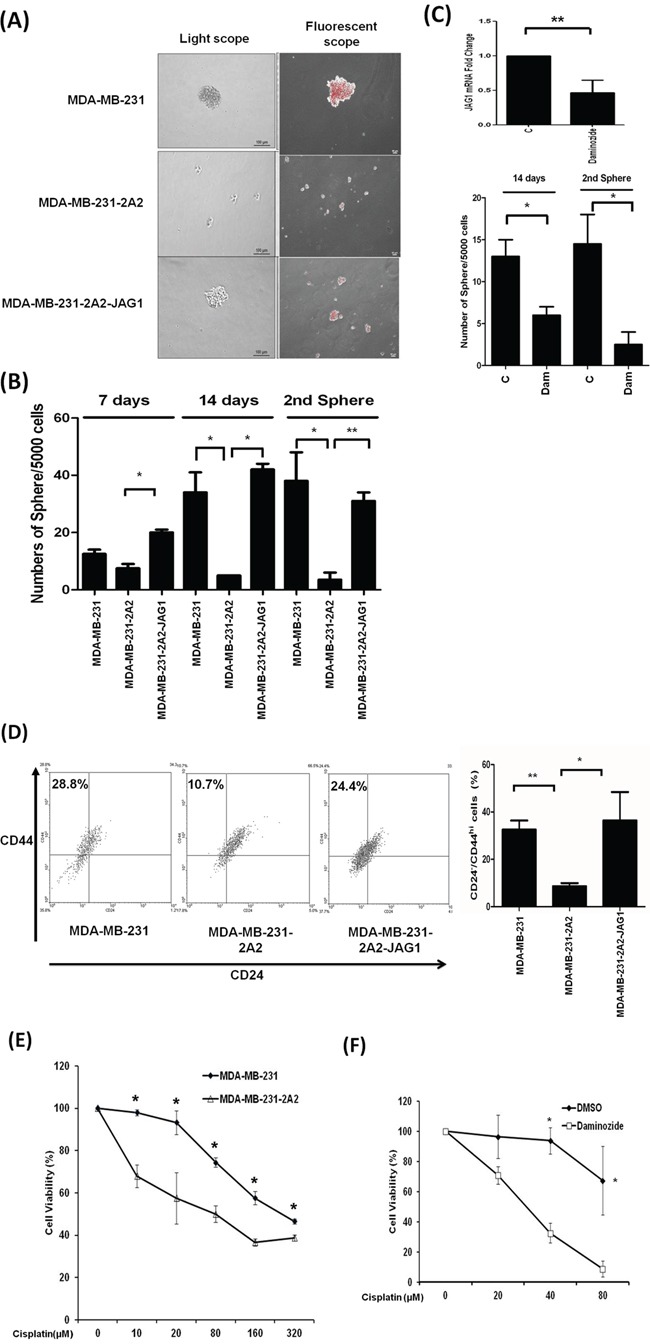
Knockdown of KDM2A reduced tumorsphere formation and enhanced cisplatin sensitivity **A.** Cells were labeled with PKH26 and subjected to sphere formation assay. The morphology of tumorsphere and the retention of PKH26 on cells were detected by light and fluorescent microscope respectively. **B.** The sphere formation ability of breast cancer cell lines with KDM2A knockdown or JAG1 overexpression was assayed by culturing cells in low attachment plates. For the second sphere formation, the spheres collected at day 14 were dispersed to single cell by continuous pipetting and then re-seeded into the low attachment plate for another round of sphere formation assay. **C.** The effect of a KDM2A inhibitor daminozide (Dam) on the expression of *JAG1* and the sphere formation of MDA-MB-231 cells. **D.** The population of CD24^−^/CD44^hi^ cells in breast cancer cell lines with KDM2A knockdown or JAG1 overexpression was determined by flow cytometry. A typical figure was shown in the left panel and the results from three independent assays were shown in the right panel. **E.** The cytotoxic effect of cisplatin on MDA-MB-231 cells and KDM2A-depleted MDA-MB-231-2A2 cells measured by MTT assay. **F.** The viability of MDA-MB-231 cells pre-incubated without or with the KDM2A inhibitor daminozide followed by treatment of different concentrations of cisplatin. *p<0.05, **p<0.01 and ***p<0.001.

The NOTCH1 signaling pathway plays a crucial role in the maintenance of stemness of normal and cancer stem cells. Breast cancer cells with stem-like properties are slow-dividing and relatively quiescent within a proliferating population. These cancer stem cells could retain the lipophilic dye PKH26 for a long time after labeling [16]. We stained MDA-MB-231 cells with PKH26 and then seeded the cells onto low attachment plates for sphere formation assay. The fluorescent intensity was retained in the tumorspheres indicating the cancer stem-like properties of the cells (Figure 5A). Knockdown of KDM2A reduced the sphere formation of MDA-MB-231 cells and ectopic expression of JAG1 fully reversed the reduction of the tumorspheres in MDA-MB-231-2A2 cells (Figure 5B). We collected the tumorspheres after 14 days and repeated the sphere formation assay. As shown in Figure 5B, the second sphere formation was also inhibited by KDM2A depletion. Similarly, a KDM2A chemical inhibitor daminozide reduced *JAG1* expression and strongly inhibited the sphere formation of MDA-MB-231 cells (Figure 5C). Breast cancer stem cells express high CD44 and are negative for CD24. We found that the population of CD24^−^/CD44^hi^ cells was reduced in MDA-MB-231-2A2 cells and ectopic expression of JAG1 reversed the reduction (Figure 5D). Another characteristic of breast cancer stem cells is the resistance to chemotherapeutic drugs. We showed that KDM2A-depleted cells are highly sensitive to cisplatin (Figure 5E). In addition, KDM2A inhibitor daminozide significantly enhanced the cytotoxic activity of cisplatin to MDA-MB-231 cells (Figure 5F). These data suggested that inhibition of KDM2A reduces stemness and chemoresistance of breast cancer cells.

### KDM2A increases the stemness regulator SOX2 via JAG1

**Figure 6:**
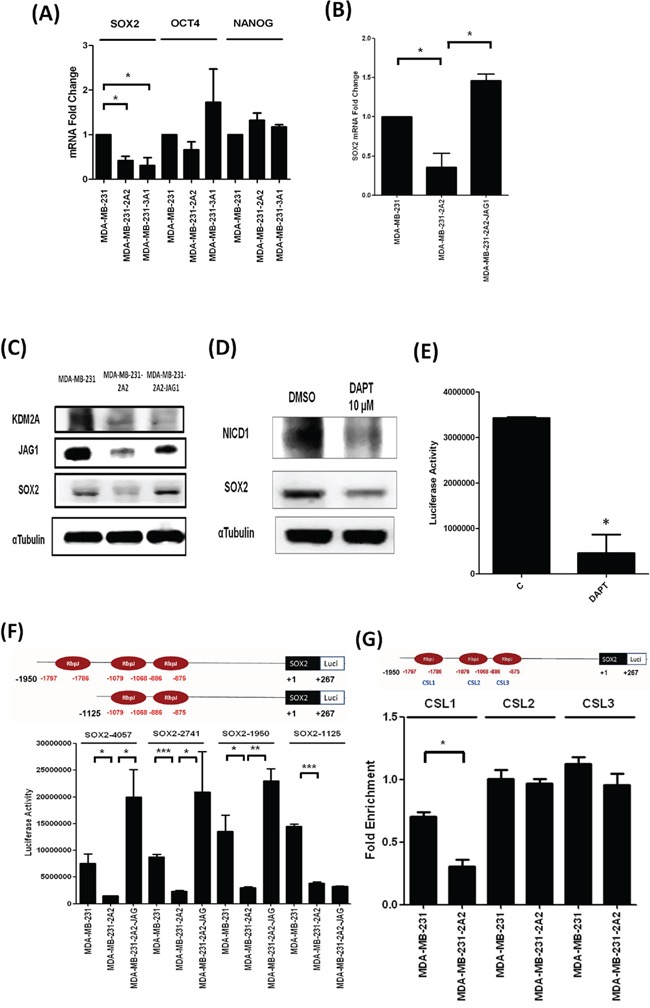
KDM2A increased cancer stemness via SOX2 **A.** Expression of three stemness transcription factors in MDA-MB-231 cells and two KDM2A-depleted clones was studied by qRT-PCR. **B.** *SOX2* expression in breast cancer cell lines with KDM2A knockdown or JAG1 overexpression was studied by qRT-PCR. **C.** The protein level of KDM2A, JAG1 and SOX2 in breast cancer cell lines with KDM2A knockdown or JAG1 overexpression was investigated by Western blot analysis. **D.** MDA-MB-231 cells were treated without or with theγ-secretase inhibitor DAPT and the level of NICD and SOX2 protein was studied by Western blot analysis. **E.** Cells were transfected with the human SOX2 promoter-luciferase construct and were incubated without or with theγ-secretase inhibitor DAPT (10μM) for 24 h. Luciferase activity was determined as described in Materials and Methods. **F.** The scheme showed the *SOX2* gene promoter region (upper panel) and the effect of KDM2A knockdown and JAG1 overexpression on the *SOX2* promoter activity was studied by determining luciferase activity. **G.** Three *SOX2* promoter constructs with mutations in specific CSL sites were trasnfected into cells and the luciferase activity was determined to identify the specific CSL binging site involved in the regulation of *SOX2* promoter activity by KDM2A. *p<0.05, **p<0.01 and ***p<0.001.

We next investigated the effect of KDM2A depletion on stemness regulators including *SOX2*, *OCT4* and *NANOG* and our data showed that only *SOX2* was down-regulated by KDM2A knockdown (Figure 6A). Ectopic expression of JAG1 completely rescued the reduction of SOX2 in MDA-MB-231-2A2 cells (Figure 6B). Western blot analysis demonstrated that overexpression of JAG1 does not affect KDM2A level while it reversed the expression of *SOX2* suggesting JAG1 is a downstream mediator of KDM2A to upregulate SOX2 (Figure 6C). Similar results were also found in KDM2A-depleted SKBr3 cells (Figure 4A and 4B). NOTCH activation induced by JAG1 leads to the generation of a NOTCH intracellular domain (NICD) which complexes with Maml and Rbpj to activate downstream transcriptional target genes via the Rbpj binding sites [17]. We found high levels of NICD and SOX2 proteins in MDA-MB-231 cells (Figure 6D). This data is consistent with the result of a previous study showing the high level of NICD in MDA-MB-231 cells [18]. Inhibition of NOTCH activation by a γ-secretase inhibitor DAPT simultaneously decreased these two proteins and also attenuated *SOX2* promoter activity in MDA-MB-231 cells (Figure 6D and 6E). Bioinformatics analysis revealed three Rbpj binding sites within the −1797/−875 region of human *SOX2* gene promoter. Our data showed that the transcriptional activity of three *SOX2* promoter constructs containing the -4057/+267, -2741/+267 and -1950/+267 promoter region are reduced in KDM-depleted cells which could be fully reversed by JAG1 overexpression (Figure 6F). Interestingly, JAG1 could not rescue the downregulation of the -1125/+267 promoter construct which lacks the Rbpj site located at the −1797/−1786 promoter region suggesting this site is important for the activation of *SOX2* expression by JAG1. Mutation of this site reduced the transcriptional activity of the -1950/+267 promoter construct (Figure 6G). However, the basal activity of the -1125/+267 promoter construct is still significantly reduced in KDM2A-depleted cells. It is possible that KDM2A modulates *SOX2* expression via multiple transcription regulators. Our results suggested that KDM2A upregulates JAG1 to promote NOTCH activation which directly activates the transcription of *SOX2* gene in breast cancer cells.

### Inhibition of KDM2A attenuates tumor angiogenesis in vitro and in vivo

**Figure 7:**
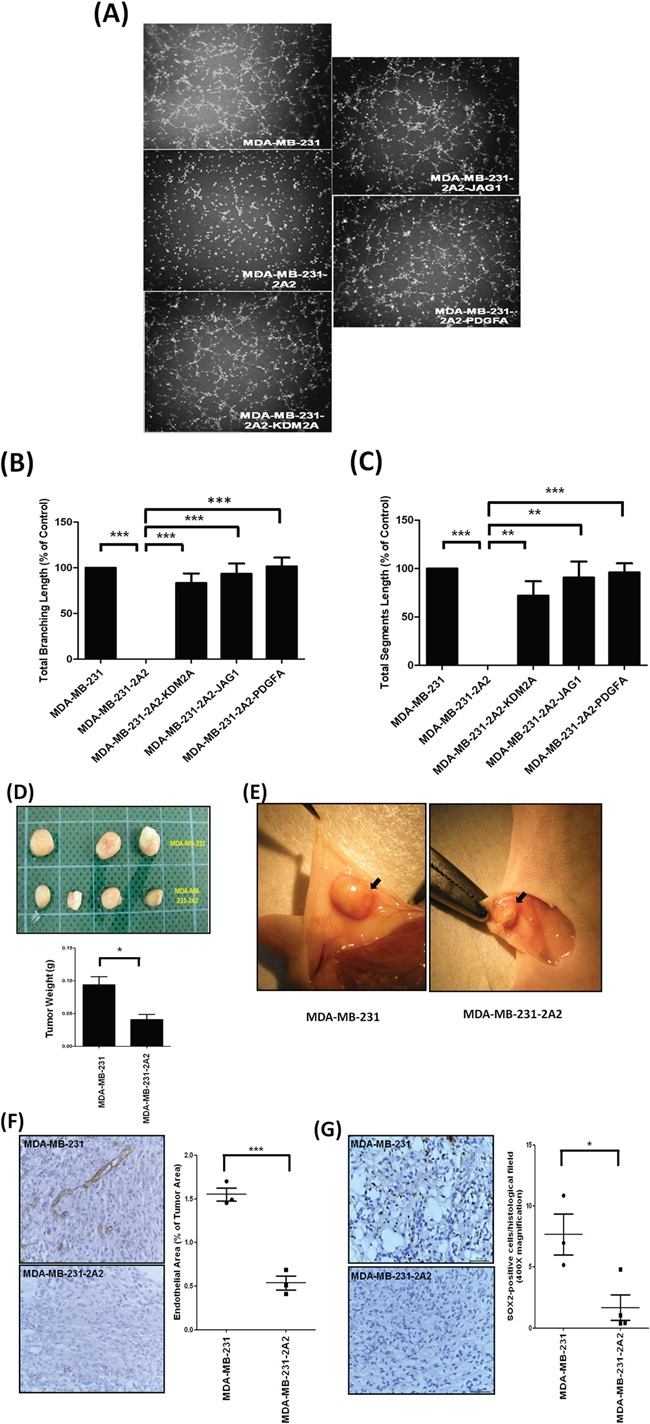
Inhibition of KDM2A attenuated tumor angiogenesis in vitro and in vivo **A.** Tube formation of EA.hy926 human endothelial cells co-cultured with breast cancer cell lines with KDM2A knockdown or overexpression of various pro-angiogenic genes. **B.** Total branching length of tubes formed by EA.hy926 human endothelial cells under co-culture conditions was analyzed by the ImageJ software. **C.** Total segments length of tubes formed by EA.hy926 human endothelial cells under co-culture conditions was analyzed by the ImageJ software. **D.** Weight of the tumors generated by subcutaneous injection of MDA-MB-231 or MDA-MB-231-2A2 cells. **E.** Typical feature showed angiogenesis of the MDA-MB-231 or MDA-MB-231-2A2 tumors. **F.** Typical picture showed the immunohistochemical staining of the CD31-positive blood vessels in the tumors (left panel) and the quantified results were shown in the right panel. **G.** Typical picture showed the immunohistochemical staining of the SOX2-positive cells in the tumors and the quantified results were shown in the right panel. *p<0.05, **p<0.01 and ***p<0.001.

Our GSEA results showed that inhibition of KDM2A is associated with reduced angiogenesis. In addition, two core-enriched genes *JAG1* and *PDGFA* are well-characterized pro-angiogenic factors. Therefore, we investigated the effect of KDM2A on tumor angiogenesis in vitro and in vivo. To test whether JAG1 expressed on breast cancer cells could induce tube formation in endothelial cells via direct contact, we co-cultured EA.hy926 endothelial cells with various MDA-MB-231 clones and found that co-culture of MDA-MB-231 cells but not MDA-MB-231-2A2 cells induced the formation of tube-like structure (Figure 7A). Ectopic expression of KDM2A, JAG1 or PDGFA in KDM2A-depleted MDA-MB-231-2A2 cells significantly increased their ability to induce tube formation and restored total branching and segment lengths (Figure 7B and 7C).

**Figure 8:**
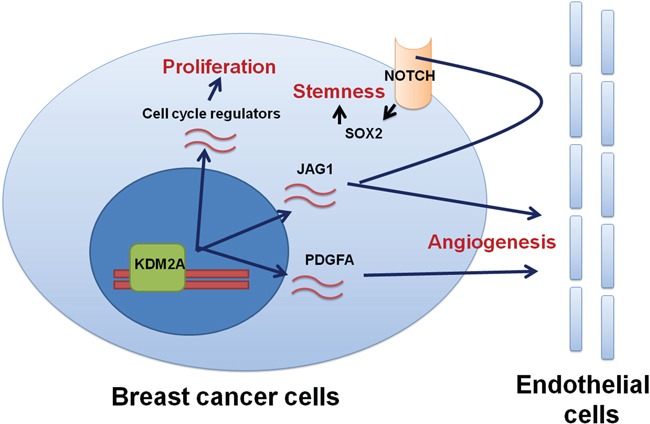
A mechanistic model shows the regulation of proliferation, stemness and angiogenesis in breast cancer by KDM2A

As shown in Figure 7D, knockdown of KDM2A reduced tumor weight by 60%. Abundant blood vessels were detected around the tumors generated by injection of MDA-MB-231 cells (Figure 7E). In addition, immunohistochemical staining showed strong CD31-positive blood vessels in the MDA-MB-231 tumors suggesting intense angiogenesis in these tumors (Figure 7F). Conversely, angiogenesis was reduced in the MDA-MB-231-2A2 tumors (Figure 7F). We also found SOX2-positive cancer cells were higher in the MDA-MB-231 tumors when compared to the MDA-MB-231-2A2 tumors (Figure 7G). Figure 8 summarized the results of this study to propose a mechanistic model by which KDM2A promotes breast cancer progression.

## DISCUSSION

In this study, we provide evidence that JAG1 is a direct target of KDM2A and is important for KDM2A to promote tumor stemness and angiogenesis. Previous studies demonstrated that high expression of JAG1 is frequently found in human breast cancer and is significantly associated with poor survival [19, 20]. In addition, aberrant activation of NOTCH signaling induced by JAG1 overexpression also predicts poor clinical outcome in breast cancer patients [20, 21]. However, the molecular mechanism by which JAG1 is activated in cancer cells is largely unclear. In epithelial cells from mammary gland, kidney tubule and epidermis, transforming growth factor Δ could stimulate JAG1 expression via SMAD3 [22]. In endothelial cells, JAG1 expression is negatively regulated by chicken ovalbumin upstream promoter-transcription factor II (COUP-TFII) to control embryonic arterial-venous differentiation [23]. In early ovarian follicle development, JAG1 gene is activated by the neurotrophin-TrkB signaling pathway in oocytes [24]. A recent study demonstrated that an oncogenic transcription factor Lim domain only 2 (LMO2) increases glioma stem cells by inducing JAG1 expression [25]. In addition to transcriptional induction, JAG1 expression could be modulated epigenetically. By using MassARRAY spectrometry, Cao et al demonstrated that the methylation level of overall and majority individual CpG sites of the JAG1 gene promoter were significantly lower in breast tumor tissues than that of adjacent normal breast tissues [26]. Because histone modification and DNA methylation are highly associated during gene transcription, it is possible that histone methylation controlled by histone methyltransferases and demethylases will affect JAG1 expression. We showed for the first time that KDM2A binds to the JAG1 promoter to increase its expression in breast cancer cells. Recently, another demethylase KDM4C has also been shown to bind onto the JAG1 promoter to mediate β-catenin-dependent transcription of JAG1 and to maintain sphere-forming capacity in colon cancer cells [27]. These data suggested JAG1 is a common target of various histone demethylases to promote tumorigenesis.

To clarify how KDM2A enhances stem-like properties of breast cancer cells, we investigated the alteration of stemness markers after KDM2A depletion and identified SOX2 as a key mediator. The importance of SOX2 in cancer stem cells has been highlighted recently by two independent studies. Vanner et al demonstrated that SOX2-expressing cells isolated from medulloblastoma exhibited self-renewal ability in vitro and high tumor-forming activity in orthotopic animals, whereas SOX2^−^ cells lacked these characteristics [28]. Boumahdi et al found that SOX2^+^ cells are absent from the normal epidermis while they are robustly increased in papilloma and squamous cell carcinoma [29]. Conditional deletion of SOX2 from the epidermis significantly delayed chemical carcinogen-induced tumorigenesis. In breast cancer, expression of SOX2 was detected in early stage tumors [30]. Importantly, SOX2, but not NANOG and OCT4, is upregulated in mammosphere in culture. Inhibition of SOX2 prevented the self-renewal ability and dramatically reduced mammosphere formation. Results of our study support the notion that SOX2 is a master regulator in maintaining breast cancer stem cells and provide the first evidence that SOX2 is a downstream mediator of KDM2A to promote cancer stemness.

We also connect the KDM2A-increased JAG1 to *SOX2* activation. Our results showed that the reduction of *SOX2* in KDM2A-depleted breast cancer cells could be fully rescued by ectopic expression of JAG1 suggesting the involvement of NOTCH signaling in the regulation of *SOX2* transcription. Previously, a genome-wide analysis of in vivo Rbpj targets in the neural stem cells also revealed that *SOX2* is a potential target of NOTCH signaling [31]. In addition, JAG1 has been demonstrated to increased *SOX2* expression in the mammalian inner ear [32, 33]. However, whether NOTCH activation could stimulate SOX2 in cancer cells is still unknown. We identified three RBPJ binding sequences within the proximal human *SOX2* promoter and demonstrated that NICD specifically binds to the Rbpj site located at the −1797/−1786 region of *SOX2* promoter. Mutation of this site significantly attenuated JAG1-induced *SOX2* promoter activity (Figure 5D and 5E). However, this site is not participated in the regulation of basal *SOX2* transcription. Our results confirmed *SOX2* is also a direct transcriptional target of the JAG1/NOTCH signaling pathway in cancer cells.

In addition to increase of stemness, we also demonstrated the promotion of tumor angiogenesis by KDM2A via PDGFA and JAG1. PDGFA is a well-characterized pro-angiogenic factor in different physiological and pathological conditions including organ development, ischemia and renal diseases [34–36]. In addition, expression of PDGFA has been shown to be associated with high vascular density, lymph node metastasis and tumor recurrence in breast cancer [37, 38]. Our data indicated that PDGFA is a direct target of KDM2A and a critical mediator of KDM2A-induced tube formation of endothelial cells (Figure 6). The angiogenesis-promoting activity of JAG1 has been elegantly demonstrated by showing that JAG1-expressing head and neck cancer cells triggered NOTCH activation in co-cultured endothelial cells and promoted capillary-like sprout formation in vitro and angiogenesis in vivo [39]. In consistent with their results, we also found that JAG1-expressing breast cancer cells activated the NOTCH signaling and increased tube formation in endothelial cells. Knockdown of KDM2A in breast cancer abolished these stimulatory effects which could be reversed by JAG1 overexpression indicating JAG1 is involved in KDM2A-induced tumor angiogenesis.

Results of this study suggest KDM2A is a potential therapeutic target for breast cancer. Daminozide, a plant growth regulator, is the first KDM inhibitor selective for the KDM2/7 subfamily and inhibits KDM2A with an IC50 value of 1.5 μM [40]. Suzuki et al developed a series of hydroxamate analogues and identified compound 9 as a potent inhibitor of KDM2A, KDM7A and KDM7B [41]. Optimisation of triazolopyridine compounds yielded another selective KDM2A inhibitor, compound 35, which exhibits >30X selectivity over members of the KDM3, 4 and 6 subfamilies [42]. Very recently, a novel cell-active KDM2A inhibitor was identified from high throughput screening [43]. The antitumor activity of these compounds on orthotopic breast cancer models warrants further investigation.

In contrast to our findings, Zizwani et al suggested a tumor suppressive role of KDM2A in breast cancer [44]. Several discrepancies are discussed as following. First, the authors showed that KDM2A was stained in the myoepithelial cells while we found that KDM2A was expressed in cancer cells. When our manuscript was submitted for review, a new paper published by Tanaka et al demonstrated that KDM2A staining was detected in tumor and nontumor areas of breast cancer [45]. As shown in their figure, the KDM2A signal in tumor part was appeared in cancer cells but not myoepithelial cells. Second, similar to our results, Tanaka et al concluded that the expression of KDM2A in breast cancer remained high during carcinogenesis while Zizwani et al concluded that KDM2A expression decreases with disease progression to metastasis. Among these three studies, only our study provided clear clinicopathological association in a large cohort of patients. Therefore, we think our study is of clinical significance. More importantly, analysis of the association of KDM2A with survival of breast cancer patients in two public databases that included more than 3,500 patients also indicated that high expression of KDM2A is linked with poor survival (Figure 1D and 1E). Third, our and Zizwani's studies all pointed out the alteration of cell cycle progression after KDM2A depletion. However, the effect is very different. The discrepancy could be attributed to the knockdown strategy. In our study, we used stable clones established by antibiotic selection. The expression of KDM2A was continuously inhibited due to the constitutive expression of shRNA from the expression vector. Conversely, Zizwani et al transiently transfected siRNA to repress KDM2A in breast cancer cells. The efficiency of transient transfection may be different from assay to assay. In addition, the inhibition of KDM2A by siRNA will decrease after cell division because of the dilution of siRNA in the divided cells. More works are needed to clarify this issue.

Taken together, we conclude that KDM2A functions as an oncogene in breast cancer by upregulating JAG1 to promote stemness, chemoresistance and angiogenesis.

## MATERIALS AND METHODS

### Cell culture and reagent

MDA-MB-231 cell line purchased from the Bioresource Collection and Research Center (Taiwan) was cultured in RPMI1640 medium containing 10% fetal bovine serum (FCS). EA.hy926 cell line was kindly provided by Dr. Ming-Hing Tai (National Sun Yat-sen University, Taiwan). pLKO.shRNA-KDM2A plasmid was obtained from the National Core Facility for Manipulation of Gene Function by RNAi, miRNA, miRNA sponges, and CRISPR/Genomic Research Center (Academia Sinica, Taipei). KDM2A expression vector was obtained from Dr. Yi Zhang (Harvard University). Daminozide was purchased from Cayman (Ann Arbor, Mi, USA). Anti-KDM2A and anti-SOX2 antibodies were obtained from Abcam (Cambridge, MA, USA). Anti-JAG1 and anti-PDGFA antibodies were purchased from Santa Cruz Biotechnology (Santa Cruz, CA, USA). Anti-CD44 and anti-α-Tubulin antibodies were purchased from GeneTex Inc. (Hsinchu, Taiwan). Anti-CD24 antibody was obtained from StemCell Tecnologies Inc. (Vancouver, BC, Canada). Anti-cleaved Notch 1 (V1744) antibody was purchased from Cell Signaling Technology Inc. (Danvers, MA, USA).

### Establishment of KDM2A knockdown stable cell line

pLKO.shRNA-KDM2A plasmid was transfected into MDA-MB-231 cells with GeneIn transfection reagent (Amsbio, Cambridge, MA, USA). After transfection, the cells were cultured at 37°C in a 5% CO2-humidified atmosphere for 48 h and then subjected to antibiotic selection with 10 μg/ml puromycin. KDM2A expression of knockdown cells was detected by Western blotting and two stable clones (named as KDM-MB-231-2A2 and MDA-MB-231-3A1) were used in this study.

### Cell proliferation assay

Cells (5000/well) were seeded onto 96 wells plates. After different times, 5 μg/ml of MTT reagent was added to each well and incubated for another 4 h. The reaction was stopped with 100 μl DMSO and the absorbance was detected at 570 nm using a microplate reader.

### Human gene expression analysis

Total RNAs were isolated from MDA-MB-231 cells and two KDM2A-depleted stable clones by using an RNA extraction kit (Geneaid, New Taipei City, Taiwan). RNA samples were subjected to microarray analysis by Human OneArray v6 (Phalanx Biotech, Hsinchu, Taiwan). Data were analyzed with Rosetta Resolver System software (Rosetta Biosoftware, USA). Standard selection criteria to identify differentially expressed genes are as follows: (1) log 2 fold change ≥ 1 and P < 0.05. (2) log 2 ratios=”NA” and the differences of intensity between the two samples≥1000. Pathway analysis was analyzed with GSEA software.

### Quantitative reverse transcription-PCR analysis (qRT-PCR)

Total RNA was isolated from cells and 1 μg of RNA was reverse transcriped to cDNA. Target mRNAs were quantified using real-time PCR reactions with SYBR green fluorescein and actin was served as an internal control. cDNA synthesis was performed at 95°C for 5 min, and the conditions for PCR were 30 cycles of denaturation (95°C/45 sec), annealing (60°C/45 sec), extension (72°C/45 sec), and 1 cycle of final extension (72°C/10 min). The primers used in this study were listed in Supplementary materials.

### Western blotting

Cellular proteins were extracted from MDA-MB-231 or MDA-MB-231-2A2 cells with RIPA buffer (50 mM Tris–HCl, pH 7.4, 150 mM NaCl, 1% NP-40, 0.1% SDS, 0.5% sodium deoxycholate, 2 mM EDTA and 50 mM NaF) and the proteins were separated by SDS-PAGE. Proteins were transferred to PVDF membranes and the membranes were probed with various primary antibodies and developed by enhanced chemiluminescence reagent.

### Luciferase activity assay

pGL3-Basic-SOX2 promoter vectors (0.5μg) was transfected into cells with GeneIn reagent in 24 well plate. After 24 h, cells were collected and lysed with 50 μL of freshly diluted reporter lysis buffer (Promega). After centrifugation, 20 μL of supernatant was added to 50 μL of the luciferase assay substrate (Promega) and the luminescence of the samples were read immediately by CentroLIApc LB 962 Microplate Luminometer (BERTHOLD TECHNOLOGIES GmbH & Co. KG, Germany), in which light production was measured for 1 second.

### Chromatin immunoprecipitation (ChIP) assay

Cells were fixed with 1% formaldehyde at 37°C for 10 min and subsequently washed twice with ice-cold PBS containing protease inhibitors (1 mM phenylmethylsulphonyl fluoride, 1 μg/mL aprotinin, and 1 μg/mL pepstatin A). Cells were incubated in a lysis buffer (1% SDS, 10 mM EDTA, 50 mM Tris-HCl, pH 8.1) for 10 min on ice and sonicated to shear genomic DNA. The lysate was centrifuged for 10 min at 13000 rpm at 4°C. The supernatant was diluted in a ChIP dilution buffer (0.01% SDS, 1% Triton X-100, 2 mM EDTA, 16.7 mM Tris-HCl, pH 8.1, 167 mM NaCl, and protease inhibitors). Anti-KDM2A, anti-dimethyl H3K36, anti-trimethyl H3K36, anti-trimethyl H3K4 or non-immune (negative control) antibodies were added to the supernatant and incubated overnight at 4°C with rotation. DNA fragments were recovered and subjected to PCR amplification using specific primers for the detection of the CpG islands upstream of JAG1 or PDGFA gene transcription start site.

### Tube formation assay

PKH67-stained EA.hy926 endothelial cells were incubated with MDA-MB-231, MDA-MB-231-2A2, MDA-MB-231-2A2-JAG1 or MDA-MB-231-2A2-PDGFA cells for 8 h in 24-well plates coated with 250 μl of 10 mg/ml Matrigel (BD). The total branching length and segments length were calculated by ImageJ software.

### Sphere formation assay

Cells were cultured with DMEM/F12 medium containing B27 supplement (GIBCO), 20 ng/ml EGF and 20 ng/ml FGF2 in low-attachment 6-well plates. After 7 or 14 days, spheres were collected without pipetting and fixed with 3.7% formaldehyde. The spheres with a diameter greater than 60 μm were counted. For the second sphere formation, the spheres collected at day 14 were dispersed to single cell by continuous pipetting and then re-seeded into the low attachment plate for another sphere formation assay.

### Flow cytometric analysis of CD44^hi^/CD24^−^ cancer stem cell population

Cells were cultured with DMEM/F12 medium containing B27 supplement, 20 ng/ml EGF and 20 ng/ml FGF2 in low-attachment plates for 14 days. Cells were harvested, and incubated with anti-CD24 and anti-CD44 antibodies for 1 h at 4°C. Cells were washed and incubated with Alexa Fluor 594 anti-rabbit and Alexa Fluor 488 anti-mouse IgG antibody for another 1 h. The CD44^hi^/CD24^−^ cells were detected by flow cytometry.

### Cytotoxicity on tumor spheres

To test whether KDM2A inhibition could enhance chemosensitivity, the mammospheres generated from MDA-MB-231 cells were cultured in the absence or presence of 5μM Daminozide for 7 days in low-attachment plates. The mammospheres were collected and dispersed to single cells by continuous pipetting. The cells were seeded onto 24 well plates. After attachment, cells were treated with different concentrations of cisplatin for 24 h and viable cells were counted by using trypan blue exclusion assay.

### In vivo orthotopic animal study

MDA-MB-231 or MDA-MB-231-2A2 cells (1x 10^6^/mouse) were suspended in Hank's balanced salt solution and inoculated into the fourth mammary fat pads of 6 week-old female BALB/cAnN.Cg-Foxn1nu/CrlNarl mice. After 4 weeks, tumor-bearing animals were sacrificed and the tumors were isolated from mice. The tumor weight was measured and the statistical difference between experimental groups was evaluated by *t*-test. Tumor tissues were used for immunohistochemical study. Animal use protocol was approved by the Institutional Animal Care and Use Committee of National Health Research Institutes.

### Immunohistochemical study of clinical samples

Paraffin-embedded tissue sections of human breast cancer specimens were obtained from Chi-Mei Medical Center (Tainan, Taiwan). The slides were stained with anti-KDM2A antibody and the signal intensity was interpreted using the H-score, defined by the following equation: H-score = ΣPi (i + 1) as previously described [46], where is the intensity of the stained tumor cells (0 to 3+), and Pi is the percentage of stained tumor cells with various intensities. Tumors with H-scores greater than the median of all cases were regarded as high expression. Survival analyses for disease-specific and metastasis-free survival were performed using Kaplan-Meier plots and compared using the log-rank test. This study was approved by the Research Ethics Committee of National Health Research Institutes. Written informed consent was obtained from all patients participated in this study.

### Statistical analysis

Student *t* test was used to compare independent groups in our in vitro and in vivo experiments. Disease-specific and metastasis-free survival were performed using Kaplan-Meier plots and compared using the log-rank test. Two-tailed P values ≤ 0.05 were considered statistically significant. Statistical analysis was performed using the GraphPad Prism version 5.01 (GraphPad Software, Inc.).

## SUPPLEMENTARY MATERIALS


