# Supplementary material for: Lysine demethylase 2A promotes stemness and angiogenesis of breast cancer by upregulating Jagged1
Source: Oncotarget. 2016 Mar 25;7(19):27689–710. doi: 10.18632/oncotarget.8381 (PMC5053681; doi:10.18632/oncotarget.8381)
Supplement: Supplementary file 1 [file oncotarget-07-27689-s001.pdf]

# SUPPLEMENTARY MATERIAL

| Primer list | |
|---|---|
| Real-time PCR | |
| JAG1-forward | 5’-TGTGAAATTGCTGAGCACGC-3’ |
| JAG1-reverse | 5’-ACACAAGGTTTGGCCTC ACA-3’ |
| PDGFA-forward | 5’-GGCCAAGGTGGAATACGTCA-3 |
| PDGFA-reverse | 5’-TCACCTCACATCTGGTTGGC-3’ |
| SOX2-forward | 5’-ACACCAATCCCATC CACACT-3’ |
| SOX2-reverse | 5’-GCAAACTTCCTGCAAAGCTC-3’ |
| OCT4-forward | 5’-GTACTCCTCGGTCCCTTTCC-3’ |
| OCT4-reverse | 5’-CAAAAACCCTGGCACA AACT-3’ |
| Nanog-forward | 5’-CAAAGGCAAACAACCCACTT-3’ |
| Nanog-reverse | 5’-TCTGCTGGAGGCTGAGGTAT-3’ |
| Actin-forward | 5’-TGTTACCAACTGGGAC GACA-3’ |
| Actin-reverse | 5’-GGGGTGTTGAAGGTCTCAAA-3’ |
| Chromatin immunoprecipitation (ChIP) assay | |
| JAG1-forward | 5’-TGCTGTTGCATTTGAGGGGA-3’ |
| JAG1-reverse | 5’-CAAACCGGCCGCTGAATAGT-3’ |
| PDGFA-forward | 5’-GAGACGCTT CCTTGGGTGAG-3’ |
| PDGFA-reverse | 5’-CTCCGGATTCGGCCTTTGAA-3’ |
| CSL1 (-1797~-1786) binding site-forward | 5’-GCAGGAAGGTTGATTGGAAA-3’ |
| CSL1 (-1797~-1786) binding site-reverse | 5’-CTACCAGCCACGTTCCATTT-3’ |
| CSL2 (-1079~-1068) binding site-forward | 5’-CACGACCGAAACCCTTCTTA-3’ |
| CSL2 (-1079~-1068) binding site-reverse | 5’-GGAAAGACACAGACGGAAGG-3’ |
| CSL3 (-886~-875) binding site-forward | 5’-CACGACCGAAACCCTTCTTA-3’ |
| CSL3 (-886~-875) binding site-reverse | 5’-CTCCCTGTCCAACTCAGCTC-3’ |
